# T26. PERINATAL STRESS AND PSYCHOSIS: RESULTS FROM THE BOLOGNA FIRST EPISODE PSYCHOSIS (BO-FEP) STUDY

**DOI:** 10.1093/schbul/sby016.302

**Published:** 2018-04-01

**Authors:** Ilaria Tarricone, Marcello Lanari, Marta Di Forti, Robin Murray, Domenico Berardi

**Affiliations:** 1 Bologna University; 2 Institute of Psychiatry, King’s College London

## Abstract

**Background:**

According to the gene-environment interaction model the pathogenesis of psychosis relies on an adverse neuro-socio-developmental pathway. Perinatal stress represents an important risk factor for the development of psychosis because it could interfere with socio-neuro-development early in life. We aim to investigate the correlation of perinatal risk factors with the onset of psychosis.

**Methods:**

Case-control – incidence study. Patients (and their mothers) were eligible if they presented for the first time with first episode psychosis at the Bo West CMHC between 2002 and 2012. The Bo West CMHC serves a catchment area of about 200,000 people. The controls were recruited in the same catchment area and study period.

**Results:**

42 patients and 26 controls and their mothers were included. Adjusted logistic regression showed that psychosis onset was significantly associated with: stressful situations during pregnancy; lower level of maternal physical health before or during pregnancy; use of anti-inflammatory drugs during pregnancy; low level of maternal education.

**Discussion:**

The results of our study suggest that stress during perinatal period increases the risk of developing psychosis. More attention should be given to the containment of perinatal stress and the prevention of its adverse effects on mother and child mental health.

Tarricone I, Mimmi S, et al.. First-episode psychosis at the West Bologna Community Mental Health Centre: results of an 8-year prospective study. Psychol Med. 2012 Nov;42(11):2255–64. PubMed PMID: 22404368

